# Male Gender Identity and Reversible Hypokalemic Hypertension in a 46,XX Child with 11-Beta-Hydroxylase Deficiency Congenital Adrenal Hyperplasia

**DOI:** 10.7759/cureus.5248

**Published:** 2019-07-26

**Authors:** Alpesh Goyal, Hiya Boro, Rajesh Khadgawat

**Affiliations:** 1 Endocrinology, All India Institute of Medical Sciences, New Delhi, IND

**Keywords:** congenital adrenal hyperplasia, cah, 11β-hydroxylase deficiency, hypokalemia, hypertension, gender identity, reversible hypertension

## Abstract

Steroid 11-beta-hydroxylase deficiency is a relatively rare form of congenital adrenal hyperplasia (CAH). We describe the case of a 46,XX child, reared as a male, who first presented to us at the age of three years with features of peripheral precocity and hypokalemic hypertension. Based on the clinical and biochemical profile, a diagnosis of 11-beta-hydroxylase deficiency CAH was established, and physiological glucocorticoid replacement was begun. Both hypertension and hypokalemia improved with glucocorticoid supplementation, and at eight years of age, antihypertensives were successfully withdrawn. Regression of left ventricular hypertrophy was also noted at this time. In keeping with the male gender identity, the child underwent hysterectomy, oopherectomy and breast reduction surgery at 13 years of age. We conclude that both hypertension and end-organ damage due to 11-beta-hydroxylase CAH may get reversed following optimal glucocorticoid treatment. Detailed genital examination at birth may help in early diagnosis of this rare disorder, thereby preventing the deleterious consequences of longstanding mineralocorticoid excess.

## Introduction

Congenital adrenal hyperplasia (CAH) due to 11-beta-hydroxylase deficiency (online Mendelian inheritance in man (OMIM) #202010) is an autosomal recessive disorder of glucocorticoid biosynthesis, characterised by features of androgen and mineralocorticoid excess. It is the second most common form of CAH after 21-hydroxylase deficiency and accounts for 5-8% of all CAH cases [1]. The estimated incidence of this rare disorder is about one in 100,000 to 200,000 live births. However, its occurrence is much higher in certain ethnic populations and geographical locations in North Africa and the Middle East, where consanguinity is common. The disorder is caused by a homozygous or compound-heterozygous mutation in the *CYP11B1 *gene located on chromosome 8q24.3, which codes for the 11-beta-hydroxylase enzyme. The enzyme catalyzes the conversion of 11-deoxycortisol to cortisol and 11-deoxycorticosterone (DOC) to corticosterone in zona fasciculata of the adrenal cortex. An enzyme defect leads to low cortisol and elevated adrenocorticotrophic hormone (ACTH), with consequent adrenal hyperplasia and shunting of steroid precursors into the androgen synthesis pathway, resulting in hyperandrogenism. Accumulation of DOC and other steroid precursors results in low renin hypertension, a finding seen in two-thirds of patients at the time of diagnosis [1,2].

## Case presentation

**Figure 1:**
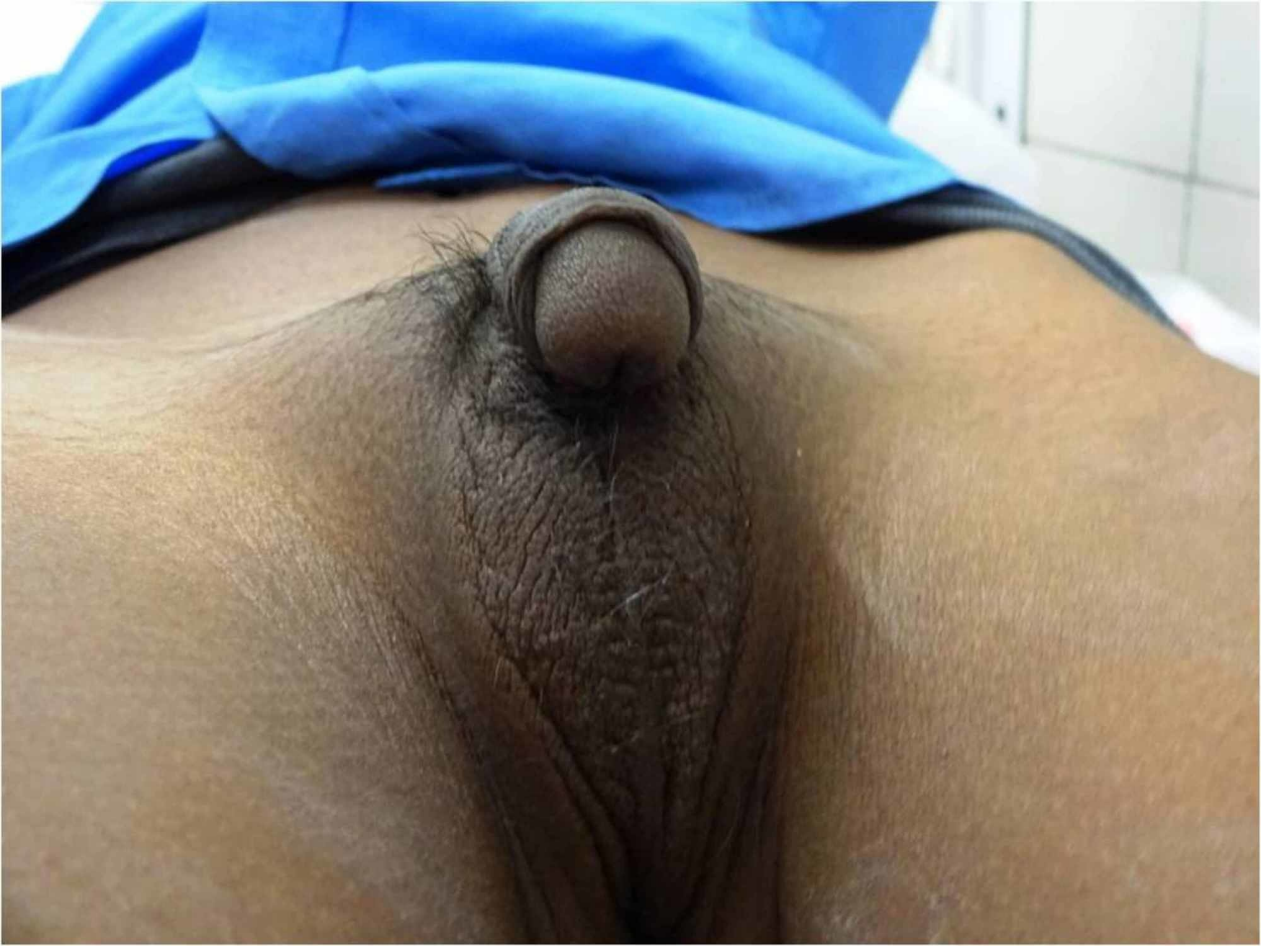
Clinical photograph showing fused labioscrotal folds with absent gonads, ventral chordee, and penoscrotal hypospadias

A 3-year-old child was brought to us with the complaints of the passage of urine from the undersurface of the phallus and downward phallic curvature since birth. The parents also noted the abnormal appearance of pubic hair and excessive aggressiveness in the child since the age of two years. The child was fifth in birth order and was born out of non-consanguineous parentage. There was no history of a salt-wasting crisis, antenatal maternal virilisation or intake of any hormonal preparation by the mother. The child was raised as a male by the family and was given a name consistent with the same. In retrospect, the parents admitted that the child was darker in complexion compared to other siblings, and the darkening increased further two months before the presentation. There was no family history of early neonatal deaths, ambiguous genitalia or infertility. On examination, the child had a supine blood pressure of 144-154/90-94 mmHg (>99th centile for age and height, consistent with the diagnosis of hypertension). Examination of genitals revealed local hyperpigmentation, enlarged phallus with ventral curvature, penoscrotal hypospadias, fused labioscrotal folds, and single urethral opening (Prader 4 virilisation) (Figure 1).

Anthropometry revealed a weight of 17 kg, a height of 101 cm (more than 75th centile) with midparental height of 169 cm for a male child (between 25th and 50th centile) and 156 cm for a female child (less than 5th centile).

Investigations revealed hypokalemia, primary adrenal insufficiency with elevated sex steroids, and elevated 11-deoxycortisol (Table 1).

**Table 1:** Baseline investigations of the patient TSH: Thyroid stimulating hormone, LH: Leuteinizing hormone, FSH: Follicle stimulating hormone, ACTH: Adrenocorticotropic hormone, DHEAS: Dehydroepiandrosterone sulfate *11-deoxycortisol was assayed using Liquid Chromatography-Tandem Mass Spectrometry (LCMS/MS). All other hormone assays were performed using Electrochemiluminescence Immunoassay (ECLIA).

Parameter	Value	Normal values/Comments
Hemoglobin (g/dL)	12.2	12-15
Serum Urea/creatinine (mg/dL)	20/0.4	20-40/0.3-1.2
Serum Na^+^/ K^+^ (meq/L)	144/2.4	135-145/3.5-5.5
Serum T4 (µg/dL)	8.35	4.5-12.5
Serum TSH (µIU/mL)	4.06	0.27-4.2
Serum LH/FSH (IU/L)	<0.01/0.127	Prepubertal
Serum Cortisol 8 am (µg/dL)	3.88	6.2-19.4
Plasma ACTH 8 am (pg/mL)	1200	7.2-63.3
Serum DHEAS (µg/dL)	69.98	Pubertal value
Serum Testosterone (ng/mL)	3.01	Pubertal value
Serum Cortisol (µg/dL) 60 min. post Synacthen 250 µg	2.39	N: >18 post stimulation
Serum 11-deoxycortisol (ng/dL)* 60 min. post Synacthen 250 µg	2350	N: 78-237 post stimulation

Karyotype (GTG banding) was 46,XX. Bone age by Greulich and Pyle method was advanced at 11 years and six months. Imaging of abdomen and pelvis revealed enlarged hyperplastic bilateral adrenal glands, normal Mullerian structures, normal bilateral ovaries, and opening of the genital tract into the urethra. 2D echocardiography revealed concentric left ventricular hypertrophy (LVH). A dilated fundus examination was, however, normal.

Differential diagnosis for hypertension with hypokalemia includes primary hyperaldosteronism (adenoma, hyperplasia, familial), CAH (11-beta-hydroxylase, 17-alpha-hydroxylase deficiency), syndrome of inappropriate mineralocorticoid excess due to mutation in gene encoding 11-beta-hydroxysteroid dehydrogenase type 2 (11β-HSD2) enzyme, ectopic Cushing syndrome due to overwhelming of 11β-HSD2 enzyme, DOC producing tumour, glucocorticoid resistance syndrome, renal artery stenosis and Liddle syndrome [3,4]. Based on the clinical and biochemical features, a diagnosis of 46,XX CAH due to 11-beta-hydroxylase deficiency was established in our patient. Unfortunately, a genetic analysis could not be done due to financial constraints.

**Figure 2:**
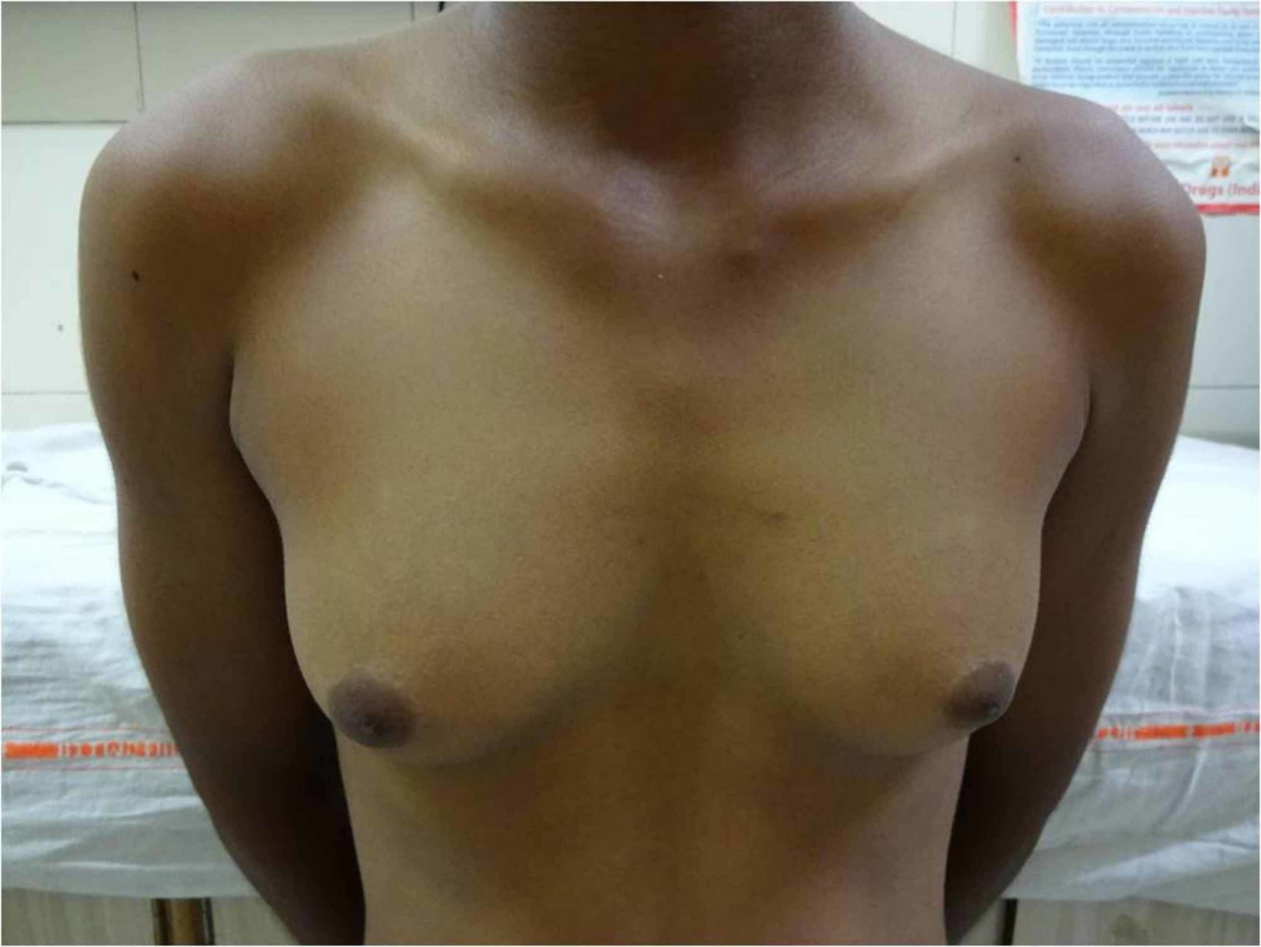
Clinical photograph showing Tanner stage 3 breast development

The child was started on hydrocortisone supplementation with stress dosage education; hypokalemia was corrected with oral potassium supplements and hypertension controlled using a combination of amlodipine and spironolactone. On regular outpatient visits, the child’s growth velocity, bone age, blood pressure, and potassium were monitored. We found that the child could be gradually weaned off potassium supplements with the maintenance of normal serum potassium levels. The antihypertensive requirement also reduced gradually, leading to gradual withdrawal and complete cessation of all antihypertensives by the age of eight years. A 2D echocardiography repeated at this time revealed regression of LVH. The child was raised as a male by the family and had a male gender identity, which was confirmed with psychological tests. The child was subsequently lost to follow-up and again presented to us at the age of 13 years with complaints of progressive bilateral breast enlargement over the past four years (Figure 2).

There was no complaint of cyclical abdominal pain or hematuria. At this stage, the child was re-admitted and underwent definitive hysterectomy and bilateral salpingo-oophorectomy. This was followed by subcutaneous mastectomy and chordee correction. The child is now planned for a second stage surgery for hypospadias correction, following which testosterone replacement therapy will be initiated.

## Discussion

The diagnosis of CAH, provided by the window of abnormal genitalia at birth, was missed in this child, accounting for late presentation with severe hypertension and end-organ damage. Most females with virilising CAH are diagnosed at birth with ambiguous genitalia, while the diagnosis is delayed in males, who have normal genitalia at birth and present in early childhood with precocity [5,6]. Rarely, females with CAH and severe intrauterine androgen excess are raised as a male and present late, similar to our case [7-10].

Khattab et al. described the largest series of 11-beta-hydroxylase CAH, with 108 patients from 11 countries [2]. In this study, authors have concluded that severe virilisation in 46,XX girls is more common in 11-beta-hydroxylase CAH, compared to 21-hydroxylase CAH. This is evident from the finding that about 70% of 46,XX patients with 11-beta-hydroxylase CAH had Prader 4 or Prader 5 external virilisation score at presentation. The severe virilisation may imply a higher possibility of male gender assignment at birth, as seen in our case. About 60 percent of patients in the study described had hypertension; however, the authors reported a weak correlation between hypertension and DOC excess. The authors also noted that 11-deoxycortisol was the most robust marker for the biochemical diagnosis of 11-β hydroxylase CAH.

Both hypertension and hypokalemia resolved with optimal glucocorticoid replacement in our patient. The severity of hypertension in 11-beta-hydroxylase CAH varies from severe refractory cases requiring bilateral adrenalectomy to milder cases, improving with steroid supplementation. Glucocorticoid therapy results in feedback inhibition of pituitary ACTH secretion, thus improving hypertension and its end-organ manifestations. Similar to the index patient, Onyiriuka et al. and Isiavwe et al. described the remission of hypertension and end-organ damage with glucocorticoid supplementation [11,12]. Notably, antihypertensive requirement persisted despite optimal glucocorticoid therapy in cases reported by Valsalan et al. and Tossati et al. [13,14]. In a systematic review and meta-analysis of 48 cases with CAH who underwent bilateral adrenalectomy (both 21-hydroxylase and 11-beta-hydroxylase), severe hyperandrogenism, iatrogenic Cushing syndrome or both were found to be the most common indication for surgery [15]. Of the five patients with 11-beta-hydroxylase CAH undergoing bilateral adrenalectomy for refractory hypertension, three had complete remission of hypertension following the surgery, while the other two required persistent antihypertensive treatment.

## Conclusions

11-beta-hydroxylase deficiency CAH is a rare autosomal recessive disorder of glucocorticoid biosynthesis, which presents with features of mineralocorticoid and androgen excess. Delayed diagnosis, as in our case, may put such individuals at an increased risk of end-organ damage due to uncontrolled hypertension. A detailed genital examination and directed investigations at birth may go a long way in preventing these undesirable consequences. Hypertension and even end-organ damage in the setting of 11-beta-hydroxylase CAH may reverse with appropriate glucocorticoid replacement therapy. It is important to remember that patients with 46,XX CAH and severe androgen excess may identify themselves as males, highlighting the importance of psychological tests to confirm gender identity before embarking upon surgical intervention.
